# Managing Interruptions to Improve Diagnostic Decision-Making: Strategies and Recommended Research Agenda

**DOI:** 10.1007/s11606-022-08019-w

**Published:** 2023-01-25

**Authors:** Jennifer F. Sloane, Chris Donkin, Ben R. Newell, Hardeep Singh, Ashley N. D. Meyer

**Affiliations:** 1 Center for Innovations in Quality, Effectiveness and Safety, Michael E. DeBakey VA Medical Center and Department of Medicine, Baylor College of Medicine, Houston, TX USA; 2 School of Psychology, University of New South Wales Sydney, Kensington, Australia; 3 Ludwig Maximilian University of Munich, Munich, Germany

## Abstract

Interruptions are an inevitable occurrence in health care. Interruptions in diagnostic decision-making are no exception and can have negative consequences on both the decision-making process and well-being of the decision-maker. This may result in inaccurate or delayed diagnoses. To date, research specific to interruptions on diagnostic decision-making has been limited, but strategies to help manage the negative impacts of interruptions need to be developed and implemented. In this perspective, we first present a modified model of interruptions to visualize the interruption process and illustrate where potential interventions can be implemented. We then consider several empirically tested strategies from the fields of health care and cognitive psychology that can lay the groundwork for additional research to mitigate effects of interruptions during diagnostic decision-making. We highlight strategies to minimize the negative impacts of interruptions as well as strategies to prevent interruptions altogether. Additionally, we build upon these strategies to propose specific research priorities within the field of diagnostic safety. Identifying effective interventions to help clinicians better manage interruptions has the potential to minimize diagnostic errors and improve patient outcomes.

## INTRODUCTION

Interruptions pervade our daily lives. Although interruptions can facilitate performance, it is well-known that interruptions most often negatively affect decision-making and even the well-being of the decision-maker. For example, emergency department (ED) physicians are interrupted as frequently as 7 times per hour^1^ and interruptions have been associated with a significant increase in medication errors.^2^ Prior research has found individuals fail to return to the original task 13–18% of the time after being interrupted.^3^ Interruptions during diagnostic decision-making are also inevitable and can have negative consequences. One study found that interrupted radiology residents were 12% more likely to have made diagnostic errors in their final reports compared to when they were not interrupted^4^ and others have found that interrupted emergency physicians may take longer to report final diagnoses compared to those who were not interrupted even when accuracy is not affected.^5^ Furthermore, the use of Electronic Health Records (EHRs) is now a central part of diagnostic decision-making. Although EHRs can provide easier access to patient data and timeliness of documentation, EHRs may also result in information overload and disrupt providers’ workflow.^6^ For example, one study concluded that the interaction between the EHR design and interruptions can “lead to reduced physician-EHR efficiency levels.”^7^^[p270]^ Evidence-based strategies and interventions can not only reduce interruptions, but also mitigate the negative impact of inevitable and unavoidable interruptions, such as the risk of inaccurate or delayed diagnosis.

**Figure 1:**
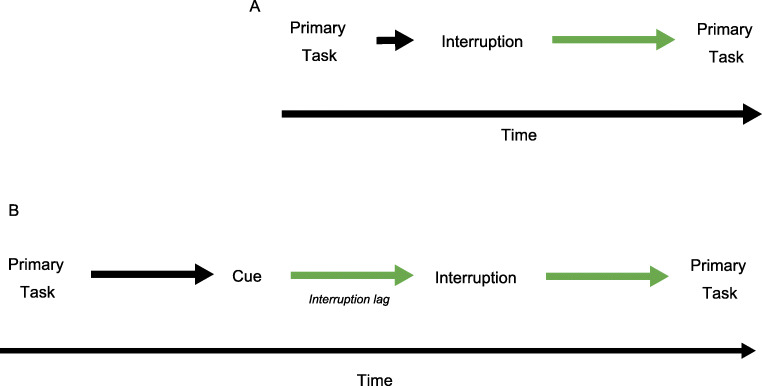
**A**, **B** Visual representation of the interruption process. Green arrows represent where interventions can be implemented. Figure 1**B** is adapted from Trafton et al. (2003).^9^

Additional knowledge, evidence, and frameworks are needed to inform the development and implementation of such strategies. This is a challenging task because diagnosis itself is a complex process and research specific to interruptions on diagnostic decision-making is limited. While there is ample research on the effects of interruptions within other realms of health care (e.g., medication administration in the intensive care unit), most are observational or non-experimental studies. As Coiera plainly puts it, “Simply counting more interruptions is unlikely to be helpful.”^8^^(p358)^ Rather, we need to focus on testing and implementing effective interventions that will create environments conducive to overcoming interruptions or mitigating their effects. Using a modified model of interruptions^9^ (see Fig. 1), we propose strategies empirically tested in the fields of health care^10–13^ and cognitive psychology^9,14–16^ that can lay the groundwork for additional research to mitigate the effects of interruptions during diagnostic decision-making. Accordingly, we use these strategies to propose specific research priorities.

## MODEL OF INTERRUPTIONS

We define interruptions as a secondary task that, to perform, requires an individual to stop working on their primary, or original, task. The primary task can be resumed only once the interruption is completed. Importantly, clinicians often juggle many ongoing tasks and may face multiple, simultaneous interruptions and the primary task with current highest priority may change over time. Examples of interruptions in diagnosis range from electronic alerts diverting a clinician’s attention in the middle of test ordering, to a request to sign paperwork that interrupts a clinician’s focus during diagnostic hypothesis generation, to a delirious patient in the room next door that requires a clinician to stop everything and go check on him/her (notably, not all of these can be inhibited).

### Conceptual Model of Interruptions

Figure 1 illustrates the interruption process as a function of time with the green arrows representing where strategies can be implemented. Sometimes interruptions are urgent, requiring individuals to switch tasks immediately. In these situations, there is no time to prepare between the primary task and the interruption (Fig. 1A), so strategies must be implemented post-interruption. However, interruptions are often non-urgent (Fig. 1B). Here, a cue (a signal to the individual that they should prepare to switch tasks) can intercede. For example, when a physician receives a text notification, the vibration or the sound of the notification is a cue that they will soon need to switch tasks, but they may have time to prepare if they believe the interruption is not urgent or if they are in the middle of another important task. In these situations, individuals have more flexibility and, thus, it is possible to implement strategies either before or after the interruption.

### Other Factors that Interact with Interruptions and Diagnostic Decision-Making

There are other factors that disrupt decision-making that are not included in our model such as cognitive load. We also do not account for interactions between interruptions and these factors, such as when trying to predict how disruptive interruptions may be. For example, one review concluded that there are three main disruptive factors: duration of the interruption, complexity of the interruption, and the moment of the interruption.^17^ It is important to acknowledge that not all interruptions negatively affect behavior; while interruptions can facilitate performance on simple tasks, they often lead to errors on harder and more complex tasks/environments (e.g., tasks involving higher cognitive load).^18,19^

The impact of interruptions on diagnostic decision-making is similarly complicated by different environments (e.g., inpatient vs outpatient). For example, the dynamic, fast-paced environment with high-acuity patients being taken care of by multiple health care professionals in the ED makes it highly susceptible to both frequent and disruptive interruptions. Interruptions are also found to disrupt cognitive processes in inpatient settings,^20^ where one study found medical residents were interrupted more frequently on inpatient rotations compared to outpatient rotations.^21^ Conversely, the outpatient setting may be somewhat less prone if clinicians are able to attend to one patient at a time and in the comfort of a private room. Nevertheless, interruptions still occur in all settings, so it is imperative to understand their implications and discover strategies to manage them within different medical environments.

## STRATEGIES FOR MINIMIZING THE IMPACT OF INTERRUPTIONS VERSUS PREVENTING THEM

Table 1 presents several strategies to help manage interruptions. While this is a non-exhaustive list, we selected frequently used, empirically tested strategies that may be a good starting point for the field of diagnosis. Additionally, we identify important categories to consider, such as whether the strategy is aimed at preventing versus minimizing the impact of interruptions and whether the strategy is aimed at an individual, team, or system level. For instance, in high-risk environments (e.g., medication administration), it may be safest to implement strategies aimed to prevent interruptions at all costs, while other environments may find greater benefits from implementing strategies aimed to minimize the negative impact of interruptions. As illustrated in our conceptual model, some interventions are more appropriate to implement before an interruption occurs, whereas others are more appropriate for implementation after an interruption. Future studies can test this model by exploring environments both with and without interruptions *and* with and without interventions to not only assess the impact of interruptions on quality of care, but also measure the effectiveness of the specific strategies to decrease or prevent the negative effects of those interruptions. While the following strategies have largely been tested in other areas of health care and psychology, we propose future research questions that could help inform work to improve diagnostic decision-making in the presence of interruptions.

**Table 1:** Proposed Strategies to Prevent Interruptions and Minimize the Impact of Interruptions at Individual, Team, and System Levels

Strategy	Minimize/prevent	Individual/team/system	Existing empirical evidence	Future research questions
Mobile device and alert management	Minimize	Individual, team, or system	• A clinical communication process intervention about how to relay emergent, urgent, and non-urgent messages reduced the proportion of total interruptions from 82 to 68%.^13^	• Is clustering non-urgent messages an effective strategy to reduce the number of interruptions?
				• In a particular health care setting, is there consensus around which messages are/are not urgent?
				• Are system level interventions dealing with mobile devices sustainable and effective in the long term?
Moment to prepare	Minimize	Individual	• Interruption lags as short as 2 s aid resumption time and reduce errors in multi-stage problem solving tasks in cognitive psychology studies.^15^	• Are interruption lags effective for preventing the negative effects of interruptions in diagnosis?
				• If they are, how much time do clinicians need for a lag to be effective?
Checklists	Minimize	Individual, team, or system	• Checklists improved diagnostic accuracy in a cardiopulmonary simulator.^26^	• What type of checklists (e.g., standardized, personal) would be most effective for preventing the negative effects of interruptions on the diagnostic process?
Do-not-disturb vests	Prevent interruptions	Team or system	• Do-not-disturb vests significantly reduced the number of interruptions by 75% and the number of medication administration errors by 66%.^10^	• When should clinicians (who are tasked with diagnosing a patient) wear the vest (i.e., during which phase(s) of the diagnostic process)?
				• Should there be a limited amount of time to wear the vests?
				• Would it be beneficial to have a designated person on the team to answer queries while the clinician wearing the vest is occupied?
No interruption zones	Prevent interruptions	Team or system	• No interruption zones led to a 40.9% decrease in interruptions during medication administration in an intensive care unit.^11^	• Would designated no interruption zones for clinicians to think improve diagnostic accuracy and timeliness of a diagnosis?
				• Would no interruption zones for clinicians cause negative, unintended consequences?

### Minimizing the Impact of Interruptions

#### Mobile Device and Alert Management

Hospitals and clinicians rely on phone calls, secure text message systems, and pagers (although with decreasing use) to communicate both urgent and non-urgent messages. However, it is often difficult to discern between these, resulting in frequent, unnecessary interruptions. Indeed, one study found 68% of pages were deemed to be non-urgent.^12^ For this reason, it is essential to develop effective strategies to manage mobile devices and alerts at both individual and system levels. For instance, clinicians may be able to adjust the settings of their daily alerts (e.g., turn off non-mandatory alerts), enable features that prevent alerts from disappearing (e.g., some systems, such as the Computerized Patient Record System, offer a “Renew Alert” feature), and prioritize alerts based on urgency.^22^

One recommendation to minimize non-urgent distractions at a system level is to introduce simple rules to cluster non-urgent messages and only page the on-call resident once a certain number of messages is reached.^12^ Unfortunately, in many medical environments, it is impossible to differentiate whether a call or page is urgent or non-urgent. In these scenarios, the safe solution is to assume that everything is urgent. Returning to our conceptual model, this means clinicians need to immediately stop what they are doing to address the interruption, so there is no time for a cue or an interruption lag. Therefore, another strategy is to train staff to think about the urgency of the interruption. For example, one study implemented a triage system for interruptions, where interruptions were classified as emergent (i.e., call immediately), urgent (i.e., send a message and expect a response within an hour), or non-urgent (i.e., send a message and expect a response within the day). This intervention significantly reduced the frequency of interruptions and the proportion of non-urgent interruptions.^13^ For instance, an incoming call during morning rounds may be reserved only for an emergency, whereas a text message could be a cue for a non-urgent message, allowing clinicians to easily differentiate between urgent and non-urgent interruptions. If successfully implemented, interventions like this one will result in fewer non-urgent interruptions and allow clinicians more opportunities to utilize other interruption mitigation strategies. While it is not always possible to turn the phone off or put it on silent mode, there are strategies that can help to reduce the frequency of non-urgent alerts. These strategies have been shown to be feasible and to decrease the occurrence of interruptions,^12,13,22^ so future work should focus on their downstream effects on diagnosis.

#### Moment to Prepare

Giving someone notice of an upcoming interruption and time to prepare can offset the negative impact of interruptions.^23^ This is an example of a strategy that can be implemented before an interruption begins. Such a strategy takes advantage of a concept called interruption lag, which is the time between the cue and the interruption in Figure 1B. One model of interruptions used in cognitive science^16^ implies that interruption lags can be used to make it easier to return to a primary task in two ways: improving memory for important retrospective (e.g., “What was I doing?”) and prospective (e.g., “What was I about to do?”) information. Evidence shows that taking just 2 seconds to prepare for an upcoming interruption can improve performance on some primary tasks.^15^ Interruption lags are useful because that time can be used to think about a current task or goal, and thus create memories of what you were doing, making it less likely you will forget to complete the task after the interruption. One way to prepare for an upcoming interruption is to quickly jot down or simply think about what you are doing (e.g., about to order the specific tests to rule out a do-not-miss diagnosis). Unfortunately, a clinician may not always have time to prepare before an interruption, so effective strategies that can be implemented after interruptions, such as using checklists, are also needed.

#### Checklists

Checklists could improve recovery from interruptions because they support prospective memory for tasks that involve remembering to do something in the future^24^ (e.g., several sequential tasks of ensuring follow-up of abnormal test results). Checklists may also improve performance because they “provide an alternative to reliance on intuition and memory in clinical problem solving,”^25^ ^(p307)^ where both processes may be compromised by interruptions.^15^ Indeed, the use of a checklist resulted in improved diagnostic accuracy for medical residents who were tasked with diagnosing on a cardiopulmonary simulator.^26^

Diagnostic decision-making is an iterative process with many steps. For example, one study provides examples of checklists specific to diagnosis and includes a sequence of steps (e.g., “Obtain your own complete medical history,” “generate initial hypotheses,” “pause to reflect,” “embark on a plan,” and “ensure a pathway for follow-up”).^25^ ^(p309)^ Highlighting the “pause to reflect” step in this checklist, we encourage clinicians to take a moment after being interrupted to consider any possible biases or heuristics (i.e., mental shortcuts) that may have adversely influenced their judgment and reasoning. In medicine, this is also known as a “diagnostic time-out.” Prospective hindsight is one useful technique that can be used during this time-out, which asks individuals to imagine a future where the current diagnosis is wrong, motivating clinicians to take time to reflect and contemplate alternative diagnoses and utilize decision support tools if necessary.^25^

Additionally, this leads us to question whether interruptions are more or less likely to be deleterious at different points in the process, an idea we return to in the “Future Directions” section. While there is promising evidence for the effectiveness of checklists in medicine,^27^ more research is needed within diagnosis to better understand how checklists can impact clinical decision-making and particularly how checklists may be able to mitigate errors caused by interruptions.

### Preventing Interruptions

#### Do-Not-Disturb Vests

One popular, albeit somewhat controversial, intervention to reduce errors caused by interruptions is the use of do-not-disturb vests. Do-not-disturb vests have been found to significantly reduce the number of interruptions by 75% and the number of medication administration errors by 66%,^10^ suggesting this intervention has the potential to effectively promote patient safety. However, several studies have abandoned such interventions, fearing the do-not-disturb vests sent the wrong message that nurses should not be “bothered,”^28^ and other studies have shown while interventions like these may work in the short term, they may be unsustainable as people resort back to old habits.^29^ Additional research is needed to determine how such interventions can be adapted for diagnostic decision-making and ensure they are effective in the long-run and framed in an appropriate and positive way.

#### No Interruption Zones

A related concept to do-not-disturb vests is no interruption zones (NIZ), which may also be referred to as Medication Safety Zones or Healthcare Sterile Cockpits. Interestingly, the phrase “sterile cockpit” was coined in 1981 after multiple aviation errors were caused by distractions and simple oversights. As a result, the Federal Aviation Administration implemented the sterile cockpit rule to remove all interruptions during critical moments of a flight, such as take-off and landing. Because the sterile cockpit rule was so successful in aviation, the concept was adopted and tried in other fields, including in health care. One study that directly tested the impact of a NIZ during medication administration in an ICU found a 40.9% decrease in interruptions.^11^

Furthermore, some EHR systems have features that allow clinicians to set their availability (e.g., Epic’s Secure Chat has the following options: “Available,” “Busy,” “Do Not Disturb”). While this feature does have a Do Not Disturb option and, therefore, the potential to reduce interruptions, it does not prevent messages from being sent. Because interruptions are a safety culture issue, more research needs to be done to understand whether these features are effective and impact behavior or if clinicians will continue to send messages regardless of clinicians’ known availability.

### Implications of Preventive Interventions for Diagnostic Safety

Diagnosis is an iterative process that evolves over time and is often collaborative.^30^ Therefore, further research is needed to test whether strategies like do-not-disturb signs and NIZ can be successfully adapted and implemented into the workflow of diagnosticians because these strategies have been tested only during medication administration. Not all diagnostic decision-making is done in a “high-risk” environment, for example, compared to a nurse who is interrupted while administering medication and where an incorrect dose could have devastating consequences. Medication administration is a task with a specific beginning and an end and is usually done by one person. While these strategies may be good solutions for individual nurses on medication rounds, it may not always be feasible or safe to create sterile cockpit type environments during diagnostic decision-making. Indeed, it may be difficult to identify “critical moments” in diagnosis for when these interventions should be implemented and upon which member(s) of the team the intervention should be used for. Perhaps one such “critical moment” could be while interviewing an acutely ill patient or while interpreting diagnostic tests. Future work could help identify these critical junctures in which diagnostic decision-making should not be interrupted.

## FUTURE DIRECTIONS

One main goal of this perspective is to highlight the need for further research on the effects of interruptions within diagnostic decision-making. With additional research, we can work towards creating a more comprehensive framework describing the interruption process and identifying where interruptions may occur. It is challenging to disentangle all the factors involved in diagnostic decision-making because it is such a complex process and interruptions are only one type of cognitive burden. Nonetheless, randomization and rigorous experimental studies can enable us to assess the impact of the different strategies and interventions. Along with the specific empirical questions listed in Table 1, we also propose several general research questions that will help clinicians better manage interruptions and improve diagnostic decision-making.

- Which strategies are most effective for mitigating the effects of interruptions during the diagnostic process and are certain interventions more effective when bundled together?
- Which steps in the diagnostic process are most susceptible to interruptions and thus perhaps most likely to benefit from interruption mitigation strategies?
- How are interventions to prevent or mitigate interruptions affected by team composition or size?
- Should we focus on trying to prevent interruptions or accept that interruptions are unavoidable during diagnosis and focus on implementing strategies to minimize the negative impacts?

## CONCLUSIONS

While existing strategies can reduce the number of interruptions and minimize their negative impact in certain safety contexts, evidence for the effects of these interventions in diagnostic decision-making is scant. Interruptions are complex and one strategy implemented successfully in certain settings may not work for other individuals and environments. Factors to consider when designing and implementing strategies to manage interruptions during diagnostic decision-making include moment and duration of interruption and type of intervention (e.g., those listed above which prevent or mitigate the impact of the interruptions). This perspective recommends additional research to understand which interventions will be most effective. Despite the limited research of the impact of interruptions on diagnosis to date, several recommended interventions can be adapted and tested to help support or improve diagnostic decision-making. Identifying effective interventions to help clinicians better manage interruptions has the potential to minimize diagnostic errors and improve patient outcomes.
